# Real-Time Monitoring of the Cytotoxic and Antimetastatic Properties of Cannabidiol in Human Oral Squamous Cell Carcinoma Cells Using Electric Cell-Substrate Impedance Sensing

**DOI:** 10.3390/ijms232415842

**Published:** 2022-12-13

**Authors:** Chien-Chu Huang, Shao-Chih Chiu, Shih-Chi Chao, Heng-Yi Liao, Shiao-Pieng Lee, Chun-Chung Huang, Der-Yang Cho

**Affiliations:** 1 Department of Obstetrics and Gynecology, China Medical University Hospital, Taichung 406040, Taiwan; 2 Graduate Institution of Biomedical Sciences, China Medical University, Taichung 406040, Taiwan; 3 Translational Cell Therapy Center, China Medical University Hospital, Taichung 406040, Taiwan; 4 Department of Medical Research and Education, Lo-Hsu Medical Foundation, Lotung Poh-Ai Hospital, Yilan 265501, Taiwan; 5 Institute of Oral Sciences, Chung Shan Medical University, Taichung 40201, Taiwan; 6 Graduate Institute of Life Sciences, National Defense Medical Center, Taipei 114201, Taiwan; 7 Division of Oral and Maxillofacial Surgery, Department of Dentistry, School of Dentistry, Tri-Service General Hospital, National Defense Medical Center, Taipei 11460, Taiwan

**Keywords:** CBD, electric cell–substrate impedance sensing, oral squamous cell carcinoma cells

## Abstract

Cannabidiol (CBD) is an active natural compound that is extracted from *Cannabis sativa*. Previous studies show that CBD is a nonpsychotropic compound with significant anticancer effects. This study determines its cytotoxic effect on oral cancer cells and OEC-M1 cells and compares the outcomes with a chemotherapeutic drug, cisplatin. This study has investigated the effect of CBD on the viability, apoptosis, morphology, and migration of OEC-M1 cells. Electric cell–substrate impedance sensing (ECIS) is used to measure the change in cell impedance for cells that are treated with a series concentration of CBD for 24 h. AlamarBlue and annexin V/7-AAD staining assays show that CBD has a cytotoxic effect on cell viability and induces cell apoptosis. ECIS analysis shows that CBD decreases the overall resistance and morphological parameters at 4 kHz in a concentration-dependent manner. There is a significant reduction in the wound-healing recovery rate for cells that are treated with 30 μM CBD. This study demonstrates that ECIS can be used for in vitro screening of new chemotherapy and is more sensitive, functional, and comprehensive than traditional biochemical assays. CBD also increases cytotoxicity on cell survival and the migration of oral cancer cells, so it may be a therapeutic drug for oral cancer.

## 1. Introduction

Taiwan has a substantially higher incidence of oral cancer than other countries in the world. The risk of oral cancer is increased by behaviors including smoking, consuming alcohol, and chewing betel nuts [1,2]. People who use cigarettes, alcohol, and betel nuts have a 123 times higher chance of acquiring oral cancer than those who do not, according to epidemiological research conducted in 1995 by Professor Ge Yingqin at Kaohsiung Medical University [3,4]. Consuming too many carcinogens can lead to persistent inflammation [5,6] and abnormal malignant cell growth, which can result in cancer invasion or metastasis to the distal area. Oropharyngeal and oral tissues can both develop oral cancer. Squamous carcinoma, verrucous carcinoma, salivary gland cancer, and malignant melanoma are examples of cancer cell types with regard to histology. Squamous cell carcinoma and epidermoid cells are the most prevalent cell types [7,8].

Oral cancer has a poor prognosis and is invasive [9]. It can spread to the neck, jaw, chin, and upper and middle neck lymphatic system. Oral cancer is treated surgically, as is cervical lymph node removal. Surgical limitations require postoperative chemotherapy or radiation [10,11]. Patients need regular follow-up exams to prevent cancer from spreading. Metastatic oral cancer has poor prognosis [12]. Intravenous, topical, oral, and intraperitoneal chemotherapy are available. The circulatory system distributes this systemic therapy. It is used to eliminate any remaining cancer cells after surgery to slow disease progression, relieve symptoms, and stop metastasis. Target therapy and immunotherapy are two recent new oral cancer treatments [13,14]. To cure cancer, target therapy employs monoclonal antibodies, such as cetuximab, which block the extracellular ligand-binding domain of the receptor, and also tyrosine kinase inhibitors [15]. Immunotherapy is one of the more recent developments that appears promising, at least as an adjuvant therapy. This newer cancer therapy treatment modality could be combined with surgery, chemotherapy, and radiotherapy [16]. However, the current standard of care is chemotherapy, surgery, and radiation therapy. Chemotherapy inhibits the synthesis of DNA, RNA, and proteins, slowing cancer cell growth. By stopping the cell cycle or increasing tumor apoptosis, it boosts surgery, radiation therapy, and immunotherapy [13,17]. This study used cisplatin to simulate therapy. This study aims to induce apoptosis or prevent oral cancer metastasis. Apoptosis is a natural process that, in contrast to necrosis, does not result in inflammation [18]. Many apoptosis pathways have been identified [19]. Previous research used biochemical assays to distinguish between malignant and normal phenotypes before regulating cancer cell development or death [18,20]. Finding new cancer drugs that induce apoptosis is crucial, as is inducing it during treatment. In this study, the anticancer potential of cannabidiol was examined (CBD).

Several natural compounds, such as CBD, are holding a promising effect of anticancer treatment. CBD is extracted from Cannabis sativa. The formula for the compound is C_21_H_30_O_2_. CBD is a natural compound with a molecular weight of 314.47 and is one of the main active ingredients of Cannabis sativa [21]. CBD has anti-inflammatory and anti-ischemic effects and is used to treat neuropathic pain and has an antitumor effect. CBD and Δ-9-tetrahydrocannabinol (THC), the two main substances found in cannabis plants, each have various pharmacological and therapeutic effects on the human body. CBD does not have any of the psychoactive effects of Δ-9-tetrahydrocannabinol (THC), whereas THC has both psychoactive and addictive qualities [22,23,24]. Although the molecular mechanism underlying CBD’s anticancer activity is not well explored, the bulk of research demonstrates that it prevents cancer cells from proliferating by inducing apoptotic signaling [25,26]. According to previous studies, CBD alters signaling pathways crucial to the growth and spread of cancer, preventing the continuation of the cell cycle and reducing cell migration [27,28]. Animal studies have shown that CBD is efficient at halting the development of malignancies [22,23]. Additionally, it is said to trigger apoptosis in cancer cells by activating caspase pathways [29]. CBD may be useful in treating cancer because it causes a significant inhibition of tumor growth, angiogenesis, and metastasis in various cancer models [30,31,32]. Currently, CBD is mostly consumed in some of the European countries and America as a health supplement [33]. Therefore, CBD may be of use for cancer therapy. However, it is still unclear whether CBD exhibits antitumorigenic activity in oral squamous cell carcinoma cells (OSCCs). This study determines the mechanisms that cause CBD-induced cell death in OCSCs and determines the synergistic efficacy of combining chemotherapy agents with CBD. It also provides in vivo evidence that CBD reduces the growth of head and neck tumors as an effective cytotoxic agent against OSCCs. These results show that CBD may target cancer stem cells and reduce chemoresistance, so an experiment is conducted to determine the mechanism for its anti-oral-cancer effect against cancer stem cells and to increase its chemosensitivity to give more effective treatment. Recent studies show that it exhibits an anticancer effect [34] and has low toxicity [35], so it is suitable for cancer therapy.

In order to examine cell activity in vitro, electric cell–substrate impedance sensing (ECIS) is applied. For adherent cells that are grown on gold arrays, ECIS examines the complex impedance spectrum. ECIS measures the morphological alterations adherent cells undergo in response to various stimuli under particular physiological and pathological circumstances [36,37]. By monitoring changes in impedance over time, ECIS is most frequently used to measure the attachment and division of cells in addition to the barrier function of a cell monolayer [38,39]. This makes the ECIS system suitable for cytotoxicity and cell migration measurements. Its unique multifrequency conducted prior can also be used to perform a more detailed analysis of the overall resistance value measured in order to understand the minor variations in the micromotion of cells [40].

This study examines how the morphology and motility of OEC-M1 cells, a line of OSCCs, alter in response to various CBD ECIS concentrations. The cellular responses of OEC-M1 cells are assessed using annexin V/7-AAD staining tests and alamarBlue cell viability assays, two common cytotoxicity techniques. Calculating morphological parameters, such as the junctional resistance between adjacent cells (Rb) and the average distance between the basolateral cell surface and the substrate, requires comparing experimental data for OEC-M1 cells that cover an electrode with the calculated values for an appropriate cell-electrode model (h) [41,42]. This method provides a more accurate evaluation of morphological changes caused by apoptosis.

## 2. Results

### 2.1. Effects of CBD and Cisplatin on Cell Viability of OEC-M1 Cells

To investigate the anticancer properties of CBD on oral cancer, we first assess its cytotoxicity on gingival squamous cell carcinoma cells, OECM-1. Cisplatin, a conventional chemotherapy drug, was used as a positive control in the present study to further confirm the clinical application potential of CBD.

To determine the anticancer properties of CBD on oral cancer, cytotoxicity to gingival squamous cell carcinoma cells, OECM-1 was determined. Cisplatin, which is a conventional chemotherapy drug, was used as a positive control for this study to confirm the potential of CBD for clinical application.

**Figure 1:**
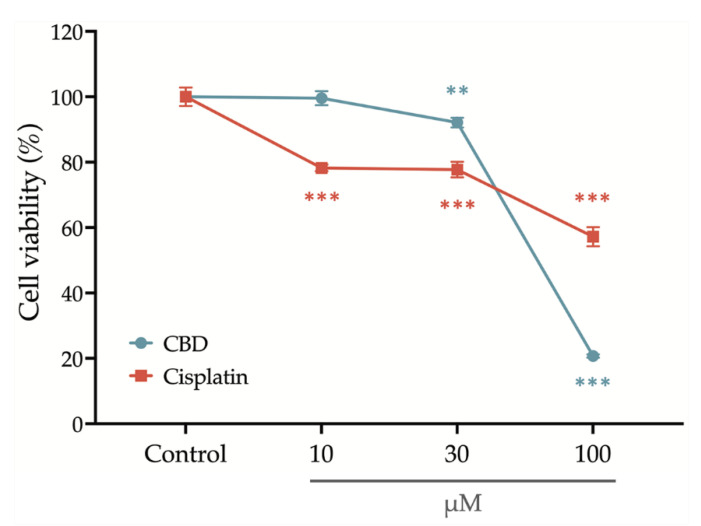
Cytotoxic effect of CBD and cisplatin on the viability of OEC-M1 cells. After the treatment with 10, 30, and 100 μM CBD (blue line) and cisplatin (red line) for 24 h, the viability of OEC-M1 cells was determined using an alamarBlue assay. Untreated cells with an equivalent percentage of DMSO were used as the control group. All data are represented as mean ± standard error mean (SEM) for at least three independent experiments. ** *p* < 0.01; *** *p* < 0.001 compared with the control group.

After treatment with a series concentration of cisplatin and CBD for 24 h, the OEC-M1 cells’ viability was determined using an alamarBlue assay. Figure 1 shows that cell viability is slightly decreased after treatment with 30 μM CBD and is significantly reduced if the concentration of CBD is increased to 100 μM. Treatment with cisplatin significantly decreases cell viability at an initial concentration of 10 μM, but the cell viability remains at 60% if the concentration is increased to 100 μM. These results show that CBD inhibits the viability of OEC-M1 cells in a concentration-dependent manner and that CBD is more cytocidal than cisplatin.

### 2.2. Effects of CBD and Cisplatin on Cell Apoptosis of OEC-M1 Cells

Phosphatidylserine (PS) translocation from the inner to the outer leaflet on a cell membrane is a crucial step in apoptosis, so the outer PS is detected using annexin V with fluorescent dye. Eventually, apoptotic cells lose the integrity of the plasma and nuclear membranes and 7-AAD enters the nucleus and binds to the nucleic acids. To determine whether CBD inhibits the viability of OEC-M1 cells by inducing apoptosis, an annexin V/7-AAD staining assay was used and both annexin V–positive (annexin V^positive^) and annexin V/7-AAD–double positive (annexin V^positive^/7-AAD^positive^) populations were considered to be apoptotic cells.

**Figure 2:**
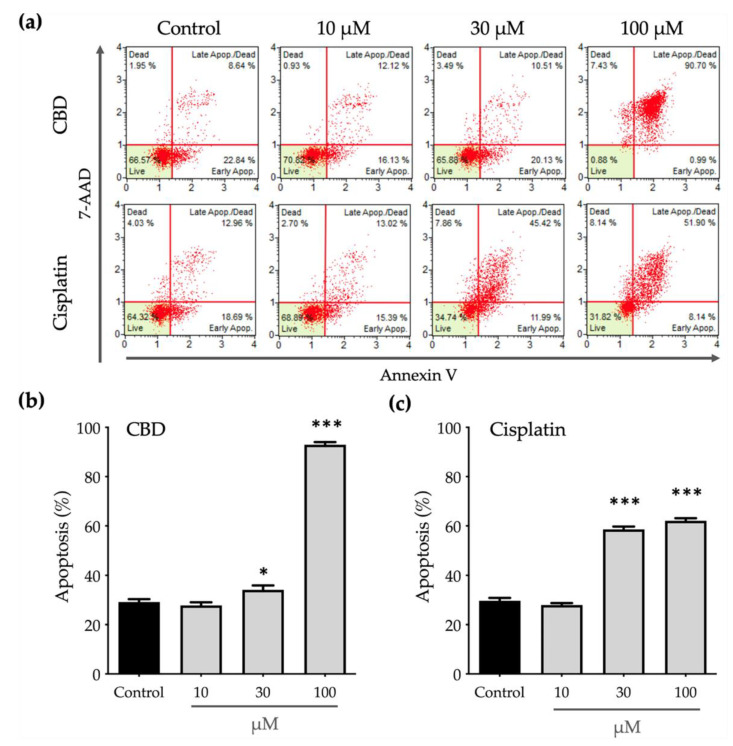
Apoptotic effect of CBD and cisplatin on OEC-M1 cells: (**a**) Apoptosis of OEC-M1 cells following treatment with CBD and cisplatin at 10, 30, and 100 μM was determined using annexin V/7-AAD staining assays. The cell populations with annexin V/PI double negative and annexin V–positive and annexin V/PI double positive are indicated as live and apoptotic cells, respectively. The statistic histogram shows the changes in the apoptotic populations after treatment with (**b**) CBD and (**c**) cisplatin for 24 h. All data are represented as mean ± standard error mean (SEM) for at least three independent experiments of (A). * *p* < 0.05; *** *p* < 0.001 as compared with the control group.

Figure 2 shows that CBD and cisplatin induce apoptosis in a dose-dependent manner. Treatment with 10 μM cisplatin significantly decreases the cell viability (Figure 1) without affecting apoptosis (Figure 2c). This decrease in cell viability may be caused by an impairment in function of the mitochondria due to cisplatin, which decreases the power of cells to reduce the alamarBlue reagent.

Apoptosis after treatment with 100 μM cisplatin produces a greater annexin V^positive^/7-AAD^positive^ population than treatment with 30 μM cisplatin (Figure 2a), which is positively correlated with lower cell viability. Similarly, to the effect on cell viability, treatment with 30 μM CBD has a significant but slight effect on cell apoptosis, as shown in Figure 2b. When the concentration of CBD is increased to 100 μM, cells almost undergo apoptosis and almost 90% of the annexin V^positive^/7-AAD^positive^ population. These results show that CBD induces cell apoptosis more efficiently than cisplatin.

### 2.3. Effects of CBD and Cisplatin on the Overall Resistance Time Course for OEC-M1 Cells

CBD has a significant effect on the viability and apoptosis of OEC-M1 cells, as confirmed by the general biochemical assays, but these do not provide comprehensive information on the time-dependent effect because there is only a single end-point measurement. The insulating nature of cell membranes allows information on morphological changes and cell–cell interactions to be obtained by measuring changes in cell impedance. ECIS, which is an impedance-based cellular assay, was used to measure the effect of CBD and cisplatin on cell morphology and the behavior of OEC-M1 cells in real time.

**Figure 3:**
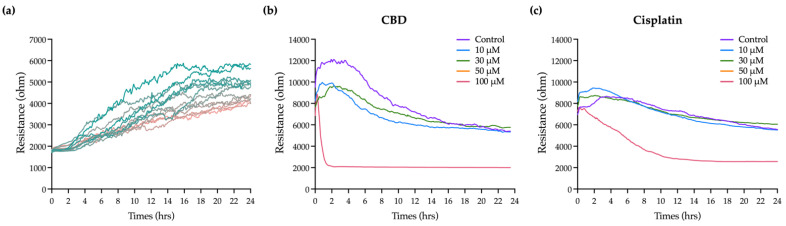
Effect of CBD and cisplatin on the integrity of the OEC-M1 cellular layer using the ECIS method: (**a**) The increased resistance time course curve shows the process for the formation of a an OEC-M1 cell monolayer. Each resistance curve is for each independent electrode containing wells. (**b**) CBD and (**c**) cisplatin were added into each ECIS well (at time 0) for 24 h of incubation. The change in resistance compared with the control group shows the integrity of the cell layer after the treatment with the drug. Each curve shows similar results for at least three independent experiments.

Figure 3a shows that the resistance in each well before adding the cells is 2 kΩ. As cells are seeded and attached and grow and expand on the electrodes, they gradually block the current flow so resistance is increased. Eventually, the resistance curve reaches a plateau at 10 to 20 h when OEC-M1 cells reach confluence. Minor fluctuations along the resistance curve are caused by cellular micromotion. To obtain the optimal results of cell resistance, current was sequentially passed through the cells at 11 frequencies. The optimal frequency of current that causes the most significant difference in resistance compared with the cell-free group is 4 kHz (data not shown). For this study, all resistance time course data were measured at 4 kHz.

Various concentrations of CBD and cisplatin were added to challenge the OEC-M1 cell layer, and changes in the overall resistance of the cell layer were measured for 24 h. Figure 3b,c shows that treatment with 10 and 30 μM CBD and cisplatin has no significant effect on the resistance, compared with the control. The cell resistance decreases significantly when it is exposed to 100 μM CBD, and the lowest resistance e (near the value of the cell-free condition, as shown in Figure 3a) is achieved within 2 h. The resistance decreases gradually for 100 μM cisplatin. These results show that ECIS gives more time kinetics information on the cytotoxicity effect than a traditional end-point assay, which is necessary to determine the starting point for the cytotoxic effect and for optimizing a therapeutic strategy.

### 2.4. Effects of CBD and Cisplatin on the Morphological Parameters for OEC-M1 Cells

**Figure 4:**
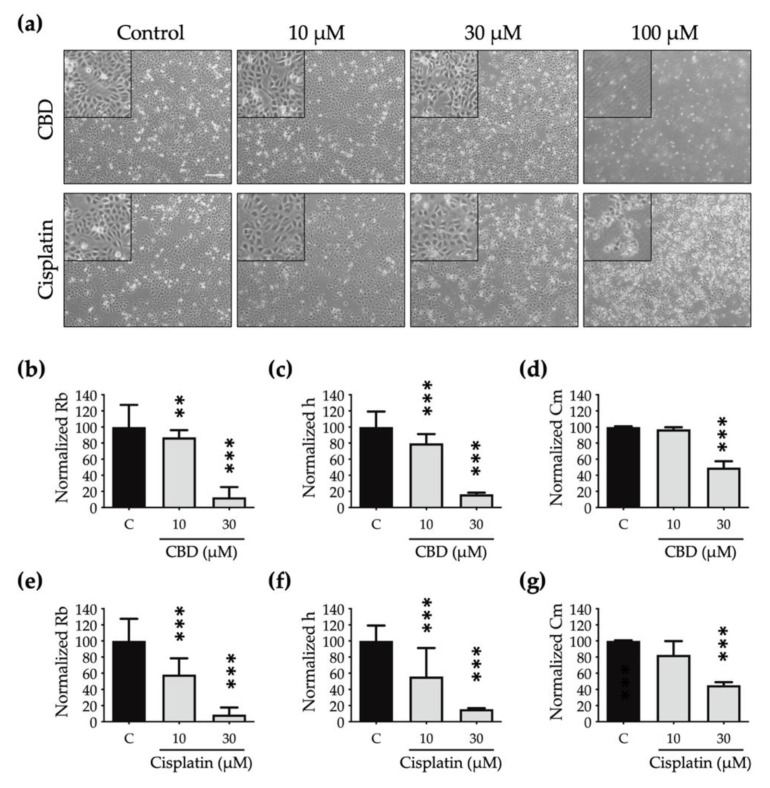
Effect of CBD and cisplatin on the cell morphology of OEC-M1 cells: (**a**) Images of OEC-M1 cells that are exposed to CBD and cisplatin at 10, 30, and 100 μM at the 24th hour from a phase-contrast microscope. Scale bar, 200 µm. The changes in the morphological parameters of the normalized junctional resistance between cells (Rb), the average cell–substrate separation (h), and the membrane capacitance (Cm) were measured using the cell-electrode model calculation for cell resistance data. The histogram shows the changes in the normalized Rb, h, and Cm values for OEC-M1 cells after treatment with (**b**–**d**) CBD and (**e**–**g**) cisplatin at 10 and 30 μM for 24 h. All data are represented as mean ± standard error mean (SEM) for at least three independent experiments. ** *p* < 0.01; *** *p* < 0.001 as compared with the control group.

A cell that dies undergoes multiple morphological changes. In Figure 4A, the phase-contrast microscopic images show that the OEC-M1 cells shrink (at 30 μM) and then detach as the concentration increases (at 100 μM) for both drugs. These are the general characteristics of cells undergoing death. However, the subtle changes in cell morphology are not clearly shown in these optical images.

To determine the effect of CBD and cisplatin on morphological changes in OEC-M1 cells, particularly in terms of cell–cell interaction and cell–substrate contact, the resistance time course data are normalized using the cell-electrode model calculation to quantify the change in the cells’ morphological parameters, including the value for the junctional resistance between cells (Rb), the average cell–substrate separation (h), and the membrane capacitance (Cm).

OEC-M1 cells almost detach after exposure to 100 μM CBD and cisplatin for 24 h, so this morphological modelling analysis is inappropriate at this concentration. Figure 4b–g shows that the Rb, h, and Cm values decrease in a dose-dependent manner after treatment with CBD and cisplatin. The values for Rb (Figure 4b,e) and h (Figure 4c,f) are less than those for cells that are treated using cisplatin, so the edge is blurred and the shape is flattened.

There is also a significant decrease in the Cm value for cells that are treated with CBD and cisplatin (Figure 4d,g), which shows that the cell membrane shrinks after treatment with both drugs and the cell volume decreases. Quantitative data for multiple cell morphological parameters analyzed by ECIS show the different toxic effects on cell shape after treatment with CBD and cisplatin.

### 2.5. Effects of CBD and Cisplatin on the Cell Migration and Micromotion of OEC-M1 Cells

**Figure 5:**
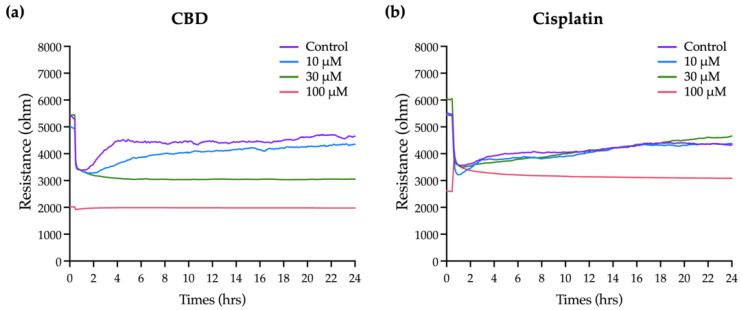
Effect of CBD and cisplatin on the migration of OEC-M1 cells. The wound area of the cell layer on the electrode is created by passing a relatively high current (at time 0, indicated by the black arrow). Subsequent resistance recovery gradients show the migration of OEC-M1 cells after exposure to (**a**) CBD and (**b**) cisplatin at 10, 30, and 100 μM. Each curve contains similar results for at least three independent experiments.

Cancer cells migrate easily, so invasion and metastasis are common. An ECIS wound-healing assay was conducted to determine the effect of CBD and cisplatin on cell migration. A high current was used to annihilate cells on the electrodes, so resistance decreases significantly, as shown in Figure 5 (black arrows). The subsequent recovery of cell impedance shows that surrounding cells migrate and cover the electrodes.

As shown in Figure 5b, when a high current is used, the resistance recovery gradients for cells that are treated with 10 and 30 μM cisplatin are similar to those for the control group. The resistance does not recover at 100 μM cisplatin, so 100 μM cisplatin completely inhibits the migration of OEC-M1 cells. As shown in Figure 5a, treatment with 10 μM CBD has no significant effect on resistance recovery. Treatment with 30 μM CBD has a limited effect on cell viability (Figure 1) but completely inhibits the resistance recovery gradient. These results show that cell migration due to treatment with 30 μM CBD is inhibited before the induction of apoptosis. Cells that are treated with 100 μM CBD are almost dead, so there is no change in resistance when a high current is used.

**Figure 6:**
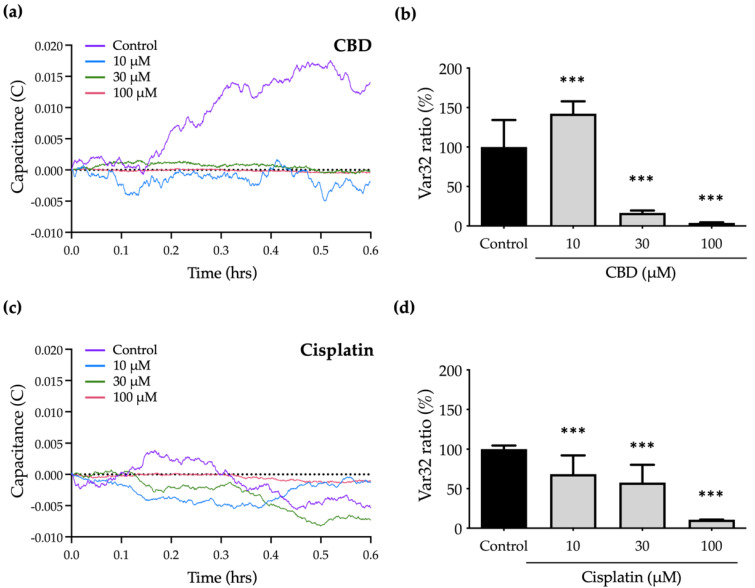
Effect of CBD and cisplatin on the micromotion of OEC-M1 cells: (**a**) The fluctuation in the capacitance (C) value time course shows the micromotion of OEC-M1 cells after treatment with (**a**) CBD and (**c**) cisplatin for 24 h. The C values are normalized and analyzed using the Var32 calculation model, and the average cell micromotion is the Var32 ratio. The histogram shows the changes in the Var32 ratio for OEC-M1 cells that are treated with (**b**) CBD and (**d**) cisplatin at 10, 30, and 100 μM. All data are represented as mean ± standard error mean (SEM) for at least three independent experiments. *** *p* < 0.001 as compared with control group.

To determine the effect of CBD and cisplatin on the micromotion of OEC-M1 cells, a numerical analysis is used to characterize the normalized capacitance (C) time courses and to calculate the Var32 values. The fluctuations in the C curve for treated cells are smaller than those for the control group because cell micromotion is inhibited. Figure 6a,c shows the significant decrease in C curves in response to treatment with CBD and cisplatin.

To quantify the cell micromotion, the normalized capacitance (C) time course values are analyzed using the Var32 calculation model. The average cell micromotion is represented as the Var32 ratio. Treatment with 10 and 30 μM cisplatin has no effect on cell migration compared with the control group (Figure 5b), but the Var32 ratio is significantly decreased, so micromotion is inhibited (Figure 6c,d). Treatment with 30 μM CBD results in a significant decrease in the Var32 ratio (Figure 6a,b), which is consistent with the complete inhibition of cell migration by CBD at the same concentration (Figure 5a). However, the Var32 ratio increases if the cells are exposed to 10 μM CBD.

These results show that treatment with 30 μM CBD significantly inhibits cell motility but has a lesser effect on cell viability, so it is a specific antimetastasis compound. The data for this study also show the advantages of ECIS for the analysis of cell migration and micromotion in that the effect of anticancer drugs on metastasis-related abilities at different levels of cellular behaviors can be determined.

## 3. Discussion

Local or regional invasion is frequently seen in oral cancer patients. Because of the abundance of vascular and lymphatic veins in this region, oral cancer has a higher potential to spread to other parts of the body. Chemotherapy and surgical resection are used to treat oral cell carcinoma that has migrated or recovered, although the mortality rate is significant.

Studies reveal that individuals receiving the usual chemotherapy medicines 5-FU [43] and cisplatin together exhibit a greater overall response, and the malignancy develops more slowly. The effectiveness of any treatment is constrained because while some patients show a particular improvement, other investigations reveal that they also exhibit clinical toxicity. There is a need for an alternate treatment approach since oral malignancies are aggressive and highly proliferative. Like traditional chemotherapy medications, pharmaceuticals must follow an inherent apoptotic route.

This investigation explores the effects of the natural substance CBD. Previous research has demonstrated that CBD therapy inhibits the invasion and migration of various cancer cells [34,44]. The concentration at which CBD has an inhibitory effect is determined by measuring cytotoxicity and cell viability using alamarBlue [34]. ECIS is a real-time label-free monitoring technique used to assess the best efficiency at various concentrations [42,45,46]. In general, OEC-M1 cells exhibit an initial stabilization phase, followed by a second stabilization phase following drug inoculation. When confluency is reached, the resistance stabilizes at 5–6 kΩ from the first phase’s low resistance of 2–3 kΩ. Because the OEC-M1 cells enlarge a little following CBD treatment, they are all more resistant to the medication than the control group. The cytoplasm shrinks and the cell shape changes when OEC-M1 cells are exposed to sufficiently high concentrations of CBD (30 μM) or cisplatin (10 μM), resulting in varying degrees of reductions in the overall resistance time courses.

Biochemical experiments were performed in order to determine the morphological alterations associated with the induction of apoptosis by anticancer substances or medications utilizing ECIS. Previous research has demonstrated that CBD exposure inhibits the invasion and migration of various cancer cells. AlamarBlue analysis of cell viability reveals the considerable inhibitory doses for both medications. There is a link between these two measurements since the profile for cell viability (%) at various concentrations is comparable to that for the Var32 ratio. This demonstrates that CBD can reduce cell micromotion when a drug’s effects remain prevalent. The change of Var32 detected by ECIS was positively correlated with its viability. Thus, in addition to demonstrating the function of CBD, it also demonstrates the potential of ECIS for novel applications in drug screening.

This study uses annexin V staining and flow cytometry to examine whether the antiproliferative action of cells following drug treatment is dependent on apoptosis. According to the results, both drugs cause an increase in the quantity of apoptotic cells that is concentration-dependent when compared with control groups. These biochemical data match with the ECIS findings. According to the results of a study, CBD has more lethal effects on OEC-M1 cells’ viability at 100 μM and migration at 30 μM when compared with cisplatin at the same concentration. This is because apoptosis causes changes in cell morphology, micromotion, and cell viability. This work also shows how to evaluate chemotherapy drugs using an impedance-based cellular assessment. ECIS measured the detailed micromotion of cells induced by drugs in a constant and real-time manner, providing a more comprehensive understanding of the overall phenomenon of cancer. Besides that, frequency analysis can monitor the important micromotion of cell behaviors, and the results of ECIS can be combined with the biochemical assays. ECIS demonstrates the possibility of developing a new noninvasive method for improving precision medicine in cancer therapy. It promotes the development of oral cancer treatments and other medical applications.

In conclusion, this study determines the effect of CBD on OEC-M1 cells. The cytotoxicity results show that CBD at higher concentrations (100 μM) increases cytotoxicity and is more likely to lead to the apoptosis of cancer cells more than cisplatin at the same concentration. ECIS is used to determine the effect of the drug on the adhesion, spread, and migration of cells. The results show that there is a linear, concentration-dependent decrease in OEC-M1 cells that are treated with CBD. Treatment with CBD at low concentrations (30 μM) completely inhibits cell migration and micromotion without affecting cell viability and apoptosis. In comparison with cisplatin, this study shows that CBD has a greater ability to inhibit metastasis and trigger apoptosis. It might work successfully as a treatment for oral cancer. We can also screen drugs more efficiently and rapidly by using the Var32 analysis method in combination with ECIS. ECIS provides a more precise measurement of experimental data and prevents operator errors by its real-time monitoring. It is promising for possible uses in new drug screening, and it might promote the development of oral cancer treatments and other medical applications.

## 4. Materials and Methods

### 4.1. Cell Culture and Reagents

OEC-M1 cells were cultured in RPMI 1640 media with 10% fetal bovine serum and 1% penicillin/streptomycin at 37 °C/5% CO_2_ and kept there at the same temperature. When a confluence level reached around 80%–90%, cells were routinely subcultured. Different CBD concentrations were introduced starting at the 24th hour after planting and continued for 48 h. One hundred percent dimethyl sulfoxide was used to dissolve the CBD (DMSO). All substances and tools were bought from Sigma-Aldrich (St. Louis, MO, USA).

### 4.2. Impedance Measurement Using ECIS

Components of the ECIS Z system were purchased from Applied Biophysics (Troy, NY, USA). This study made use of 8W1E arrays (8 wells with a 250 μm diameter gold sensing electrode). For ECIS, electrodes that are connected by a relay bank to a lock-in amplifier, which monitors in- (real) and out-of-phase (imaginary) voltages across the cell-covered electrode, were used to measure the interaction between the cell and the substrate.

An amount of 1.25 × 10^5^ cells/cm^2^ of cells were put into each well. After the cells had attained confluency 24 h after being seeded, they were given one of the four distinct anticancer medications for an additional 24 h. The impedance of each cell-covered electrode was measured at 25 different frequencies ranging from 31.25 Hz to 100 kHz in order to track changes in cell shape. A cell-free electrode and the same electrode coated with OEC-M1 cells had their impedances tested. Prior to and following cell exposure to various anticancer drug doses, the same frequency-scan measurements were made. By measuring the impedance of each electrode before and after being coated with OEC-M1 cells at 25 different frequencies, spanning from 31.25 Hz to 100 kHz, the change in cellular morphology was measured. Morphological parameters, such as junctional resistance between adjacent cells (Rb), cell–substrate distance (h), and capacitance of the cell membrane (Cm), can be determined by comparing the experimental data of the cell-covered electrode with the calculated values obtained from a suitable cell-electrode model previously described.

Additionally, a small population of cells can clearly identify a minor change in cell morphology for the micromotion measurement. Rapid time collection (RTC) in ECIS may acquire those rapid oscillatory data and use numerical analysis to reveal the micromotions of the cells in response to various coating substrates. Impedance data from each well were captured every second with a 4 kHz RTC for 0.6 h to collect 2048 points for the micromotion measurement. Variance 32 (Var32) was selected to track time-series impedance fluctuations after data collection. The 2048 data points were divided by the average value after being partitioned into 64 groups of 32 points each. The variance (the square of standard deviation) for each group was determined before being averaged across all sets.

### 4.3. Cell Viability Assay

Cell viability was assessed using the alamarBlue assay (Thermo Fisher Scientific, Carlsbad, CA, USA) according to the manufacturer’s instructions. In brief, OEC-M1 cells were plated to 24-well plates at a density of 1 × 10^4^ cells/well, and let the cell attach for 4 h. The cultured medium was replaced by a medium with CBD and cisplatin at indicated concentrations. Then, the alamarBlue reagent was directly added to each well of the 96-well plates, and cells were then incubated for another 2 h at 37 °C. The fluorescence signals of each well were measured by a Synergy H4 Hybrid microplate reader (BioTek Instruments).

### 4.4. Cell Apoptosis Assay

Cell apoptosis was determined using the Millipore annexin V/7-AAD staining technique (Billerica, MA, USA). The majority of the annexin V–positive and annexin V/7-AAD–double positive cells were found to include apoptotic cells. Cells were plated in 24-well plates at a density of 5 × 10^4^ cells per well to test the various treatments’ capacity to cause apoptosis in OEC-M1 cells. Cells were given starting doses of CBD and cisplatin at 10, 30, and 100 μM for 24 h of incubation. Following a PBS wash, trypsinization, and 10 min centrifugation at 1200 rpm, the cells were used. After centrifugation, 1 × 10^5^ cells were stained using the manufacturer’s recommended annexin V/7-AAD reagents, and flow cytometry was utilized to analyze the results using the Muse^®^ annexin V and dead cell assay technique (Millipore, Billerica, MA, USA).

### 4.5. Statistical Analysis

Statistical analysis uses a Student’s *t*-test and one-way ANOVA. All data are expressed as mean ± standard deviation. The level of significance is *p* < 0.05.

## Data Availability

Not applicable.

## References

[1] Blot W.J., McLaughlin J.K., Winn D.M., Austin D.F., Greenberg R.S., Preston-Martin S., Bernstein L., Schoenberg J.B., Stemhagen A., Fraumeni J.F. (1988). Smoking and drinking in relation to oral and pharyngeal cancer. Cancer Res..

[2] Moreno-Lopez L., Esparza-Gomez G., Gonzalez-Navarro A., Cerero-Lapiedra R., Gonzalez-Hernandez M., Domınguez-Rojas V. (2000). Risk of oral cancer associated with tobacco smoking, alcohol consumption and oral hygiene: A case-control study in Madrid, Spain. Oral Oncol..

[3] Ko Y.C., Huang Y.L., Lee C.H., Chen M.J., Lin L.M., Tsai C.C. (1995). Betel quid chewing, cigarette smoking and alcohol consumption related to oral cancer in Taiwan. J. Oral Pathol. Med..

[4] Guo S.-E., Huang T.-J., Huang J.-C., Lin M.-S., Hong R.-M., Chang C.-H., Chen M.-Y. (2013). Alcohol, betel-nut and cigarette consumption are negatively associated with health promoting behaviors in Taiwan: A cross-sectional study. BMC Public Health.

[5] Tanaka T., Tanaka M., Tanaka T. (2011). Oral carcinogenesis and oral cancer chemoprevention: A review. Pathol. Res. Int..

[6] Tanaka T., Ishigamori R. (2011). Understanding carcinogenesis for fighting oral cancer. J. Oncol..

[7] Silverman S. (2003). Oral Cancer.

[8] Neville B.W., Day T.A. (2002). Oral cancer and precancerous lesions. CA Cancer J. Clin..

[9] Bouquot J.E. (1998). Oral verrucous carcinoma: Incidence in two US populations. Oral Surg. Oral Med. Oral Pathol. Oral Radiol. Endodontology.

[10] Huang S.H. (2013). Oral cancer: Current role of radiotherapy and chemotherapy. Med. Oral Patol. Oral Y Cir. Bucal.

[11] Hartner L. (2018). Chemotherapy for oral cancer. Dent. Clin..

[12] Noguti J., De Moura C.F.G., De Jesus G.P.P., Da Silva V.H.P., Hossaka T.A., Oshima C.T.F., Ribeiro D.A. (2012). Metastasis from oral cancer: An overview. Cancer Genom. Proteom..

[13] Mohan S.P., Bhaskaran M.K., George A.L., Thirutheri A., Somasundaran M., Pavithran A. (2019). Immunotherapy in oral cancer. J. Pharm. Bioallied Sci..

[14] Naruse T., Yanamoto S., Matsushita Y., Sakamoto Y., Morishita K., Ohba S., Shiraishi T., Yamada S.I., Asahina I., Umeda M. (2016). Cetuximab for the treatment of locally advanced and recurrent/metastatic oral cancer: An investigation of distant metastasis. Mol. Clin. Oncol..

[15] Niu G., Sun X., Cao Q., Courter D., Koong A., Le Q.-T., Gambhir S.S., Chen X. (2010). Cetuximab-Based Immunotherapy and Radioimmunotherapy of Head and Neck Squamous Cell CarcinomaImaging Cetuximab Therapy of HNSCC. Clin. Cancer Res..

[16] Rapidis A.D., Wolf G.T. (2009). Immunotherapy of head and neck cancer: Current and future considerations. J. Oncol..

[17] Bold R.J., Termuhlen P.M., McConkey D.J. (1997). Apoptosis, cancer and cancer therapy. Surg. Oncol..

[18] Metzstein M.M., Stanfield G.M., Horvitz H.R. (1998). Genetics of programmed cell death in *C. elegans*: Past, present and future. Trends Genet..

[19] Alberts K., Johnson A., Lewis J., Raff M., Roberts W.P., Walter P. (2008). Chapter 18 Apoptosis: Programmed cell death eliminates unwanted cells. New York: Molecular Biology of the Cell.

[20] Majno G., Joris I. (1995). Apoptosis, oncosis, and necrosis. An overview of cell death. Am. J. Pathol..

[21] Adams R., Hunt M., Clark J. (1940). Structure of cannabidiol, a product isolated from the marihuana extract of Minnesota wild hemp. I. J. Am. Chem. Soc..

[22] Mechoulam R., Peters M., Murillo-Rodriguez E., Hanuš L.O. (2007). Cannabidiol–Recent advances. Chem. Biodivers..

[23] Zuardi A.W., Crippa J., Hallak J., Moreira F., Guimaraes F. (2006). Cannabidiol, a Cannabis sativa constituent, as an antipsychotic drug. Braz. J. Med. Biol. Res..

[24] Hampson A., Grimaldi M., Axelrod J., Wink D. (1998). Cannabidiol and (−) Δ9-tetrahydrocannabinol are neuroprotective antioxidants. Proc. Natl. Acad. Sci. USA.

[25] Velasco G., Sánchez C., Guzmán M. (2012). Towards the use of cannabinoids as antitumour agents. Nat. Rev. Cancer.

[26] Śledziński P., Zeyland J., Słomski R., Nowak A. (2018). The current state and future perspectives of cannabinoids in cancer biology. Cancer Med..

[27] Alexander A., Smith P.F., Rosengren R.J. (2009). Cannabinoids in the treatment of cancer. Cancer Lett..

[28] Salazar M., Carracedo A., Salanueva Í.J., Hernández-Tiedra S., Lorente M., Egia A., Vázquez P., Blázquez C., Torres S., García S. (2009). Cannabinoid action induces autophagy-mediated cell death through stimulation of ER stress in human glioma cells. J. Clin. Investig..

[29] McKallip R.J., Jia W., Schlomer J., Warren J.W., Nagarkatti P.S., Nagarkatti M. (2006). Cannabidiol-induced apoptosis in human leukemia cells: A novel role of cannabidiol in the regulation of p22phox and Nox4 expression. Mol. Pharmacol..

[30] Blázquez C., Casanova M.L., Planas A., del Pulgar T.G., Villanueva C., Fernández-Aceñero M.J., Aragonés J., Huffman J.W., Jorcano J.L., Guzmán M. (2003). Inhibition of tumor angiogenesis by cannabinoids. FASEB J..

[31] Blázquez C., Salazar M., Carracedo A., Lorente M., Egia A., González-Feria L., Haro A., Velasco G., Guzmán M. (2008). Cannabinoids inhibit glioma cell invasion by down-regulating matrix metalloproteinase-2 expression. Cancer Res..

[32] Qamri Z., Preet A., Nasser M.W., Bass C.E., Leone G., Barsky S.H., Ganju R.K. (2009). Synthetic cannabinoid receptor agonists inhibit tumor growth and metastasis of breast cancer. Mol. Cancer Ther..

[33] McGregor I.S., Cairns E.A., Abelev S., Cohen R., Henderson M., Couch D., Arnold J.C., Gauld N. (2020). Access to cannabidiol without a prescription: A cross-country comparison and analysis. Int. J. Drug Policy.

[34] Huang C.-C., Tung T.-H., Huang C.-C., Lin S.-Y., Chao S.-C., Chiu S.-P., Lee S.-P., Lo C.-M. (2020). Electrochemical assessment of anticancer compounds on the human tongue squamous carcinoma cells. Sensors.

[35] Astray G., Mejuto J.C., Xiao J., Simal-Gandara J. (2021). Benefits, toxicity and current market of cannabidiol in edibles. Crit. Rev. Food Sci. Nutr..

[36] Tiruppathi C., Malik A.B., Del Vecchio P.J., Keese C.R., Giaever I. (1992). Electrical method for detection of endothelial cell shape change in real time: Assessment of endothelial barrier function. Proc. Natl. Acad. Sci. USA.

[37] Arndt S., Seebach J., Psathaki K., Galla H.-J., Wegener J. (2004). Bioelectrical impedance assay to monitor changes in cell shape during apoptosis. Biosens. Bioelectron..

[38] Reddy L., Wang H.-S., Keese C.R., Giaever I., Smith T.J. (1998). Assessment of rapid morphological changes associated with elevated cAMP levels in human orbital fibroblasts. Exp. Cell Res..

[39] Keese C.R., Giaever I. (1994). A biosensor that monitors cell morphology with electrical fields. Eng. Med. Biol. Mag. IEEE.

[40] Hung Y.-H., Chiu W.-C., Fuh S.-R., Lai Y.-T., Tung T.-H., Huang C.-C., Lo C.-M. (2022). ECIS Based Electric Fence Method for Measurement of Human Keratinocyte Migration on Different Substrates. Biosensors.

[41] Balasubramanian L., Yip K.-P., Hsu T.-H., Lo C.-M. (2008). Impedance analysis of renal vascular smooth muscle cells. Am. J. Physiol.-Cell Physiol..

[42] Giaever I., Keese C.R. (1991). Micromotion of mammalian cells measured electrically. Proc. Natl. Acad. Sci. USA.

[43] Andreadis C., Vahtsevanos K., Sidiras T., Thomaidis I., Antoniadis K., Mouratidou D. (2003). 5-Fluorouracil and cisplatin in the treatment of advanced oral cancer. Oral Oncol..

[44] Go Y.Y., Kim S.R., Kim D.Y., Chae S.-W., Song J.-J. (2020). Cannabidiol enhances cytotoxicity of anti-cancer drugs in human head and neck squamous cell carcinoma. Sci. Rep..

[45] Giaever I., Keese C.R. (1993). A morphological biosensor for mammalian cells. Nature.

[46] Liu Q., Yu J., Xiao L., Tang J.C.O., Zhang Y., Wang P., Yang M. (2009). Impedance studies of bio-behavior and chemosensitivity of cancer cells by micro-electrode arrays. Biosens. Bioelectron..

